# Associations between Hypertriglyceridemia and Circulating Neutrophil Subpopulation in Patients with Dyslipidemia

**DOI:** 10.1155/2021/6695468

**Published:** 2021-05-26

**Authors:** Vadim Genkel, Ilya Dolgushin, Irina Baturina, Albina Savochkina, Alla Kuznetsova, Lubov Pykhova, Igor Shaposhnik

**Affiliations:** ^1^Department of Internal Medicine, Federal State Budgetary Educational Institution of Higher Education ^“^South-Ural State Medical University^”^ of the Ministry of Healthcare of the Russian Federation, Chelyabinsk 454092, Russia; ^2^Department of Microbiology, Virology, Immunology and Laboratory Medicine, Federal State Budgetary Educational Institution of Higher Education ^“^South-Ural State Medical University^”^ of the Ministry of Healthcare of the Russian Federation, Chelyabinsk 454092, Russia; ^3^Research Institute of Immunology, Federal State Budgetary Educational Institution of Higher Education ^“^South-Ural State Medical University^”^ of the Ministry of Healthcare of the Russian Federation, Chelyabinsk 454092, Russia; ^4^Department of Hospital Therapy, Federal State Budgetary Educational Institution of Higher Education ^“^South-Ural State Medical University^”^ of the Ministry of Healthcare of the Russian Federation, Chelyabinsk 454092, Russia

## Abstract

**Background:**

There is strong evidence to suggest that the negative influence of triglyceride-rich lipoproteins (TRLs) on atherosclerosis development and progression is at least partially mediated by their proinflammatory effects. However, the effect of hypertriglyceridemia (HTG) on the subpopulation composition of circulating neutrophils has not been studied so far. The aim of this study was to examine correlations between the level of triglycerides (TGs) and the subpopulation composition of circulating neutrophils in middle-aged patients with dyslipidemia without established atherosclerotic cardiovascular diseases (ASCVDs).

**Methods:**

Ninety-one patients with dyslipidemia, including 22 (24.2%) patients with HTG, were enrolled in the study. Phenotying of neutrophil subpopulations was performed through flow cytometry (Navios 6/2, Beckman Coulter, USA). For phenotyping of neutrophil subpopulations, conjugated monoclonal antibodies were used: CD16, PE-Cyanine7 (Invitrogen, USA); CD11b-FITC (Beckman Coulter, USA); CD62L-PE (Beckman Coulter, USA); and CD184 (CXCR4)-PE-CF594 (BD Biosciences, USA).

**Results:**

Following the correlation analysis, the TG level directly correlated with the number of circulating leukocytes (*r* = 0.443; *p* < 0.0001) and neutrophils (*r* = 0.311; *p*=0.008). HTG patients displayed a significantly high number of circulating neutrophils with CD16^hi^CD11b^hi^CD62L^hi^ and CD16^hi^CD11b^lo^CD62L^br^ phenotypes. TG levels directly correlated with the number of circulating neutrophils having CD16^hi^CD11b^hi^CD62L^hi^ and CD16^hi^CD11b^lo^CD62L^br^ phenotypes. Following the linear regression analysis, statistically significant correlations between TG levels and neutrophil subpopulations having CD16^hi^CD11b^lo^CD62L^br^ and CD16^hi^CD11b^br^CD62L^lo^CXCR4^hi^ phenotypes were established. Changes in TG levels could explain up to 19.1% of the variability in the number of studied neutrophil subpopulations.

**Conclusion:**

Among middle-aged patients without established ASCVDs, patients with HTG demonstrated a significantly higher overall number of neutrophils and neutrophils having CD16^hi^CD11b^hi^CD62L^hi^ (mature neutrophils) and CD16^hi^CD11b^lo^CD62L^br^ (immunosuppressive neutrophils) than patients with normal TG levels. The TG level was associated with an increase in the number of CD16^hi^CD11b^lo^CD62L^br^ and CD16^hi^CD11b^br^CD62L^lo^CXCR4^hi^ (ageing neutrophils) neutrophils, adjusted for the sex and age of the patients.

## 1. Introduction

Hypertriglyceridemia (HTG) is quite a commonly encountered form of lipid disorder. Thus, according to US National Health and Nutrition Examination Surveys, HTG incidence is 25% of cases in the general population and over 30% of cases in patients on statin therapy [1]. According to the Russian PROMETHEUS study (the Prevalence of Mixed Dyslipidemia and Severe Hypertriglyceridemia in the Russian Population), HTG was present in 29.2% of the Russian population [2]. However, HTG is a factor independently associated with the development of adverse cardiovascular events in various patient categories [3].

There is strong evidence to suggest that the negative influence of triglyceride-rich lipoproteins (TRLs) on atherosclerosis development and progression is at least partially mediated by their proinflammatory effects [4]. It has been established that an increase in TRL levels is associated with leukocytosis, neutrophilia, and monocytosis, as well as with enhanced expression of integrins (CD11b, CD11c, and CD18) on circulating monocytes [5, 6]. This, in turn, contributes to the development and maintenance of vessel wall inflammation [5, 7].

According to current knowledge, monocytes and their descendant macrophages play a decisive role at all stages of atherosclerotic lesion progression [8]. However, the importance of neutrophils in atherogenesis has long been underestimated, and their role in atherosclerosis has received much less attention [9]. Only in recent years has there been a significant increase in the study of neutrophils in atherosclerosis due to new discoveries in the biology of neutrophils, which led to the revision of the traditional beliefs about their functions and the heterogeneity of their population [10]. Neutrophils have come to be considered as potential therapeutic targets in the treatment of atherosclerosis [11, 12]. At different stages of the development of atherosclerosis, the significance of neutrophils of distinct subtypes may differ, which may have practical implication. Dyslipidemia as a leading risk factor for atherosclerosis can modify the subpopulation composition of neutrophils. However, the effect of HTG on circulating neutrophils has been poorly studied. Alipour et al. proved a dose-dependent two- or three-fold increase in CD11b and CD66b expression on neutrophils if incubated in a TRL-enriched emulsion [13]. However, the effect of HTG on the subpopulation composition of circulating neutrophils has not been studied so far. The aim of this study was to examine correlations between the level of triglycerides (TGs) and the subpopulation composition of circulating neutrophils in middle-aged patients with dyslipidemia without established atherosclerotic cardiovascular diseases (ASCVDs).

## 2. Materials and Methods

Patients 40–64 years of age with dyslipidemia without ASCVDs were enrolled in the study. The study included patients over 40 years of age because this is the category of patients where systematic cardiovascular risk assessment is recommended according to various clinical guidelines due to a significant increase in the risk of adverse cardiovascular events compared to younger age groups [14]. The presence of ASCVDs associated with severe extended atherosclerosis may itself have a significant impact on circulating innate immune cell pool composition, and therefore, patients with ASCVD were not included in the study [15, 16]. A necessary condition for the inclusion of patients in the study was signed informed consent. The study protocol was approved by the Ethics Committee of South Ural State Medical University (Protocol No. 10, dated October 27, 2018). Criteria for dyslipidemia were the presence of at least one abnormal finding: total cholesterol (TC) > 4.9 mmol/L; low-density lipoprotein cholesterol (LDL-C) >3.0 mmol/L; TGs >1.7 mmol/L; high-density lipoprotein cholesterol (HDL-C) <1.0 mmol/L in men, or <1.2 mmol/L in women [9]. HTG was defined as an increase in fasting TG > 1.7 mmol/L. The following conditions were used as exclusion criteria for the study: previously established ASCVDs (a history of cerebrovascular disease; coronary artery disease; peripheral artery disease; and coronary and peripheral artery revascularisation); severe hepatic and renal dysfunctions (a decrease in the glomerular filtration rate (GFR) of more than 30 mL/min/1.73 m2); malignant neoplasms; established chronic inflammatory diseases (CIDs); acute inflammatory or infectious diseases in the past 28 days; and secondary metabolic lipid disorders.

### 2.1. Laboratory Tests

All patients underwent fasting blood count tests with an automatic analyser (Medonic M16, Sweden), for which their venous blood was collected into tubes containing the K2 EDTA. The following biochemical laboratory blood parameters were obtained after fasting for at least 8 hours: TC, LDL-C, HDL-C, TG, glycated haemoglobin, and creatinine with subsequent estimated glomerular filtration rate (eGFR) calculation according to the CKD-EPI formula. The serum LDL-C level was determined by the direct method based on selective solubilisation of all classes of lipoproteins with detergents, except for LDL with subsequent LDL-C determination on an automatic analyser (BioChem Analette, USA). TG was measured in the blood serum using the colorimetric method (GPO-PAP) on an automatic analyser (BioChem Analette, USA).

**Figure 1:**
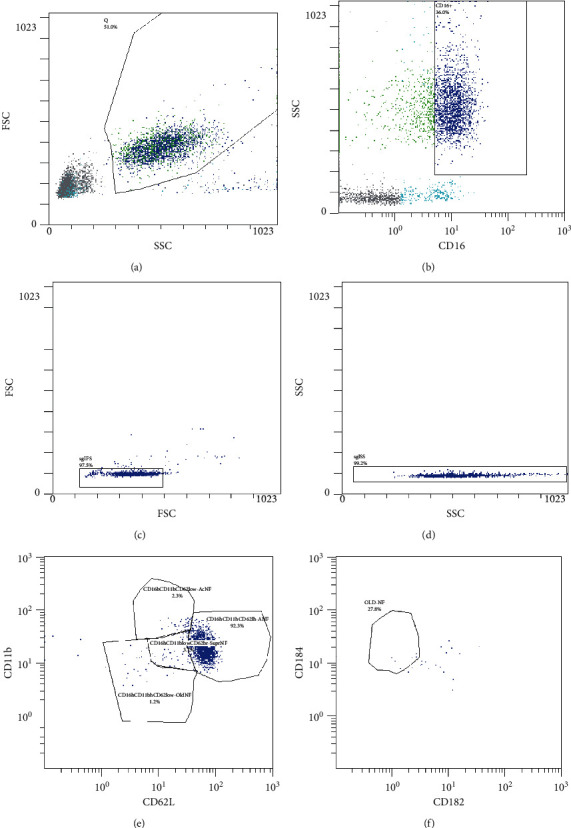
Gating strategy of flow cytometry. Sequential gating strategy for the identification of neutrophil subpopulations. Granulocytes were gated based on FSC and SSC (а). Furthermore, CD16^+^ cells were identified and single cells were gated (c–d). Identification of neutrophil subpopulations depending on CD11b and CD62 L expression (e). Identification of ageing neutrophils by the expression of CD184 and CD182 (f).

Phenotying and differentiation of neutrophil subpopulations were performed through flow cytometry (Navios 6/2, Beckman Coulter, USA). Blood was collected after fasting for at least 8 hours into K2 EDTA tubes. For phenotyping and differentiation of neutrophil subpopulations, conjugated monoclonal antibodies were used: CD16, PE-Cyanine7 (eBioscience, USA; catalog no. 25-0168-42); CD11b-FITC (eBioscience, USA; catalog no. 11-0118-42); CD62L-PE (eBioscience, USA; catalog no. 12-0629-42); CD184(CXCR4)-PE-CF594 (eBioscience, USA; catalog no. 61-9999-42); and CD182(CXCR2)-PE (eBioscience, USA; catalog no. 12-1829-42). We used whole-blood phenotyping with detection of min 30,000 events. The gating strategy of flow cytometry is represented in Figure 1.

### 2.2. Statistical Analysis

Statistical analysis of the data was performed using the Microsoft Excel software and the IBM SPSS Statistics version 18 statistical analysis package. Qualitative variables were described by absolute and relative frequencies (percentages). Quantitative variables were described with the following statistics: median (Me) and 25th and 75th percentiles (LQ, UQ) in case of nonnormal distributed variables. In order to determine correlations between the values, the Spearman correlation coefficient was used. In order to assess the significance of differences between the two groups, the Mann–Whitney test was used. Differences were considered to be statistically significant at a 0.05 significance level. In order to assess the dependence of one quantitative variable on another one, a linear regression procedure was used.

## 3. Results

Ninety-one patients with dyslipidemia, including 22 (24.2%) patients with HTG, were enrolled in the study. The patient clinical profile is presented in Table 1.

HTG patients were statistically significantly older and had higher BMI values as well. The circulating leukocyte and neutrophil number were also significantly higher in HTG patients. On the other hand, the lymphocyte and NRL number showed no significant difference between the two groups.

Following the correlation analysis, the TG level directly correlated with the number of circulating leukocytes (*r* = 0.443; *p* < 0.0001) and neutrophils (*r* = 0.311; *p*=0.008).  Table 2 shows the results of the flow cytometry.

**Figure 2:**
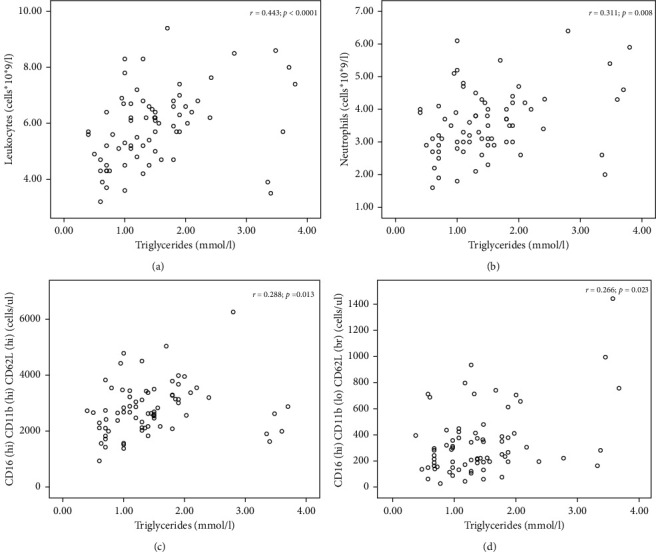
Correlation analysis demonstrating the relationship between TG and leukocytes, neutrophils, and their subpopulations.

Thus, HTG patients displayed a significantly high number of circulating neutrophils with CD16^hi^CD11b^hi^CD62L^hi^ and CD16^hi^CD11b^lo^CD62L^br^ phenotypes. The results of the correlation analysis are presented in Figure 2.

In the studied group of patients, the TG levels directly correlated with the number of circulating neutrophils having CD16^hi^CD11b^hi^CD62L^hi^ and CD16^hi^CD11b^lo^CD62L^br^ phenotypes. Moreover, it should be noted that the TG levels also directly correlated with the number of CD16^hi^CD11b^br^CD62L^lo^CXCR4^hi^ neutrophils (*r* = 0.230); however, the statistical significance of the correlation was 0.050.

**Figure 3:**
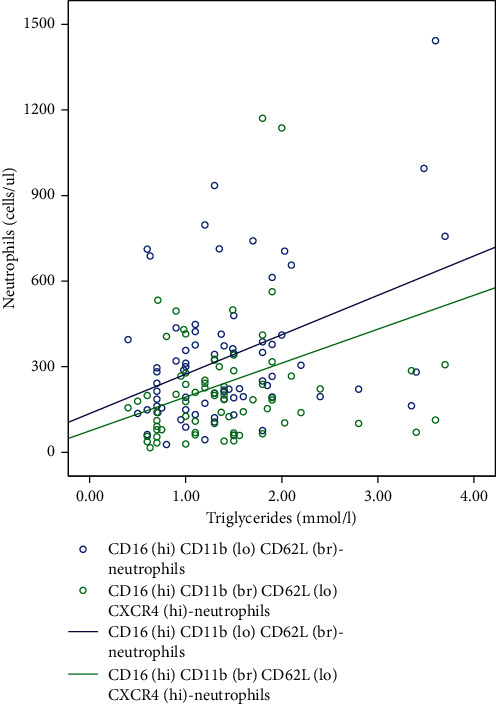
Relationship between TG and circulating neutrophil subpopulations.

In order to assess the independent effect of TG levels on the number of circulating neutrophils and their subpopulations, a linear regression analysis was performed, adjusted for a patient's sex and age (see Table 3 and Figure 3).

Following the analysis, statistically significant correlations between TG levels and neutrophil subpopulations having CD16^hi^CD11b^lo^CD62L^br^ and CD16^hi^CD11b^br^CD62L^lo^CXCR4^hi^ phenotypes were established. However, changes in TG levels could explain up to 19.1% of the variability in the number of studied neutrophil subpopulations.

## 4. Discussion

One of the results of LPL-dependent TRL catabolism is the formation of free fatty acids which, along with TRL remnants, induce the development and maintenance of an inflammatory response [17]. At present, it is assumed that the main proinflammatory effects of TRL are mediated by their influence on circulating monocytes and neutrophils [18–20]. However, information about the effect of HTG on the subpopulation composition of circulating monocytes and neutrophils is limited.

The main new findings presented by this study include the following: (1) HTG patients display a statistically significantly higher overall number of neutrophils and neutrophils having CD16^hi^CD11b^hi^CD62L^hi^ and CD16^hi^CD11b^lo^CD62L^br^ phenotypes than patients with normal TG levels; (2) following a sex- and age-adjusted linear regression analysis, an increase in TG levels was associated with an increase in CD16^hi^CD11b^lo^CD62L^br^ and CD16^hi^CD11b^br^CD62L^lo^CXCR4^hi^ neutrophils.

Correlations between TRLs and neutrophil levels were demonstrated earlier in the large population study CGPS (Copenhagen General Population Study) on 103,953 subjects [5]. The authors established a dose-dependent increase in the number of circulating neutrophils as the cholesterol remnant level increased from less than 0.5 mmol/L up to more than 2.0 mmol/L (a trend across five groups, *p* = 5 × 10^−276^). It is known that mature neutrophils in an adult patient with the absence of inflammatory diseases (in a relatively healthy state) comprise over 80% of the total circulating neutrophil pool [21]. Our study demonstrates that HTG patients are characterised by a significantly higher number of mature neutrophils, and, quite possibly, this very subpopulation is responsible for the significant increase in the overall number of neutrophils in HTG patients versus the comparison group. This is also supported by the presence of significant correlations between TG levels and the number of mature neutrophils (see Figure 1).

However, according to the linear regression data adjusted for the sex and age of patients, TG levels were independently associated with the overall number of neutrophils and the two studied subpopulations: CD16^hi^CD11b^lo^CD62L^br^ and CD16^hi^CD11b^br^CD62L^lo^CXCR4^hi^. CD16^hi^CD11b^br^CD62L^lo^CXCR4^hi^ neutrophils, also described as ageing neutrophils, are characterised by an increase in the expression of CD11b, TLR4, the ability to form neutrophil extracellular traps, and to produce active forms of oxygen [22]. It is also assumed that this subtype of circulating neutrophils by way of activating the CXCL12/CXCR4 signal pathway is characterised by an increased migration activity into inflammation sites with subsequent retention [23]. Consequently, neutrophils of this subset have high capacity to recruit into the site of atherosclerotic lesions in the vascular wall and subsequently realise their proinflammatory activity. This may be one of the pathways linking HTG and persistent inflammation of the arterial wall, promoting the atherosclerosis progression [5].

CD16^hi^CD11b^lo^CD62L^br^ neutrophils represent a subpopulation of mature neutrophils with immunosuppressive activity [24]. An increase in the number and activity of immunosuppressive neutrophils has been established during sepsis and under the influence of such stimuli as LPS and TNF [25]. It is assumed that the number of immunosuppressive neutrophils increases during a chronic systemic inflammation [26]. The immunosuppressive activity of this neutrophil subtype is predominantly realized through the inhibition of T-lymphocyte proliferation and T-cell response [27]. Moreover, the polarisation of CD4^+^ T lymphocytes changes toward Th2 lymphocytes [28, 29]. Thus, it may be concluded that the HTG effect on the subpopulation composition of neutrophils is bidirectional in nature: an increase in subpopulations has a prominent proinflammatory phenotype and, on the other hand, an immunosuppressive and anti-inflammatory phenotype. In light of the TRL effect on the development and progression of atherosclerosis, an increase in mature and ageing neutrophils is a proatherogenic factor [30–32]. The potential role of immunosuppressive neutrophils in atherogenesis has not been established yet; however, their ability to shift the polarisation of CD4^+^ T lymphocytes toward Th2 lymphocytes may suggest potential atheroprotective effects [33]. This study has a several limitations: (1) the small number of patients; (2) the difference in the number of patients included in each group; and (3) the single-center type of study.

## 5. Conclusions

Among middle-aged patients without established ASCVDs, patients with HTG demonstrated a significantly higher overall number of neutrophils and neutrophils having CD16^hi^CD11b^hi^CD62L^hi^ (mature neutrophils) and CD16^hi^CD11b^lo^CD62L^br^ (immunosuppressive neutrophils) than patients with normal TG levels. The TG level was associated with an increase in the number of CD16^hi^CD11b^lo^CD62L^br^ and CD16^hi^CD11b^br^CD62L^lo^CXCR4^hi^ (ageing neutrophils) neutrophils, adjusted for the sex and age of the patients.

## Figures and Tables

**Table 1:** Clinical and laboratory characteristics of patients.

Characteristics	NormoTG (*n* = 69)	HyperTG (*n* = 22)	Overall (*n* = 91)	*p*
Male, *n* (%)/female, *n* (%)	34 (49.3)/35 (50.7)	12 (54.5)/10 (45.5)	46 (50.5)/45 (49.5)	0.596
Age, years, Ме (LQ; UQ)	49.0 (44.0; 52.5)	54.5 (49.0; 58.7)	50.0 (45.0; 57.0)	0.031
BMI, kg/m^2^, Ме (LQ; UQ)	26.9 (24.8; 30.2)	29.7 (25.9; 32.3)	27.4 (25.2; 31.2)	0.048
Obesity, *n* (%)	18 (26.1)	10 (45.5)	28 (30.7)	0.055
Abdominal obesity, *n* (%)	39 (56.5)	16 (72.7)	55 (60.4)	0.087
Smoking, *n* (%)	16 (23.2)	5 (22.7)	21 (23.1)	0.608
T2DM, *n* (%)	4 (5.79)	2 (9.09)	6 (6.59)	0.429
Hypertension, *n* (%)	34 (49.3)	13 (59.1)	47 (51.6)	0.294
Βeta-blockers, *n* (%)	14 (20.3)	7 (31.8)	21 (23.1)	0.291
Renin-angiotensin system inhibitors, *n* (%)	15 (21.7)	9 (40.9)	24 (26.4)	0.094
Diuretics, *n* (%)	6 (8.69)	3 (13.6)	9 (9.89)	0.392
Statins, *n* (%)	19 (27.5)	9 (40.9)	28 (30.7)	0.242
Leukocytes, cells × 10^9^/l, Ме (LQ; UQ)	5.40 (4.70; 6.40)	6.50 (5.75; 7.30)	5.70 (4.75; 6.65)	0.008
Neutrophils, cells × 10^9^/l, Ме (LQ; UQ)	3.10 (2.70; 3.90)	4.00 (3.43; 4.38)	3.50 (2.90; 4.20)	0.029
Lymphocytes, cells × 10^9^/l, Ме (LQ; UQ)	1.60 (0.99; 2.10)	1.35 (1.20; 2.30)	1.50 (1.10; 2.10)	0.513
NLR, Ме (LQ; UQ)	2.08 (1.46; 3.05)	2.68 (1.71; 3.42)	2.14 (1.48; 3.50)	0.672
TC, mmol/l, Ме (LQ; UQ)	5.76 (5.01; 6.48)	6.32 (5.24; 7.17)	5.81 (5.11; 6.51)	0.134
LDL-C, mmol/l, Ме (LQ; UQ)	3.88 (3.00; 4.53)	3.34 (2.90; 4.19)	3.43 (2.90; 4.25)	0.304
HDL-C, mmol/l, Ме (LQ; UQ)	1.46 (1.20; 1.64)	1.23 (1.17; 1.38)	1.38 (1.18; 1.61)	0.061
TG, mmol/l, Ме (LQ; UQ)	1.10 (0.75; 1.40)	2.07 (1.90; 3.21)	1.35 (0.99; 1.80)	<0.0001
Non-HDL-C, mmol/l, Ме (LQ; UQ)	4.09 (3.42; 4.98)	4,93 (3.95; 5.94)	4.25 (3.59; 5.23)	0.030
Glycated haemoglobin, %, Ме (LQ; UQ)	5.60 (5.22; 6.03)	5.80 (5.35; 6.06)	5.67 (5.22; 6.05)	0.305
eGFR, ml/min/1.73 m^2^, Ме (LQ; UQ)	74.0 (62.7; 98.0)	67.5 (54.2; 84.7)	72.0 (61.0; 93.4)	0.076

NormoTG = normotriglyceridemia; HyperTG = hypertriglyceridemia; BMI = body mass index; TC = total cholesterol; HDL-C = high-density lipoprotein cholesterol; LDL-C = low-density lipoprotein cholesterol; Non-HDL-C = non-high-density lipoprotein cholesterol; eGFR = estimated glomerular filtration rate; T2DM = type 2 diabetes mellitus; NLR = neutrophil-to-lymphocyte ratio; Me = median; LQ = lower quartile; UQ = upper quartile.

**Table 2:** Subpopulations of circulating neutrophils.

Neutrophil phenotype	NormoTG (*n* = 69)	HyperTG (*n* = 22)	Overall (*n* = 91)	*p*
CD16^hi^CD11b^hi^CD62L^hi^ (mature neutrophils), cells/*μ*l, Ме (LQ; UQ)	2629 (2119; 3225)	3172 (2606; 3580)	2674 (2155; 3373)	0.029
CD16^hi^CD11b^hi^CD62L^lo^ (activated neutrophils), cells/*μ*l, Ме (LQ; UQ)	28.5 (13.0; 48.5)	24.0 (19.7; 45.2)	28.0 (15.3; 46.0)	0.670
CD16^hi^CD11b^br^CD62L^lo^CXCR4^hi^ (ageing neutrophils), cells/*μ*l, Ме (LQ; UQ)	199 (88.0; 289)	230 (132; 340)	200 (102; 309)	0.245
CD16^hi^CD11b^lo^CD62L^br^ (immunosuppressive neutrophils), cells/*μ*l, Ме (LQ; UQ)	220 (149; 373)	327 (230; 623)	258 (162; 399)	0.039

NormoTG = normotriglyceridemia; hyperTG = hypertriglyceridemia; Me = median; LQ = lower quartile; UQ = upper quartile.

**Table 3:** Linear regression analysis showing the effect of TG on neutrophil count (adjusted for sex and age).

Characteristics	*R*	*R* ^2^	*B*	95% CI for *B*		*p*
				Lower limit	Upper limit	
Neutrophils						
Triglycerides	0.349	0.122	0.445	0.153	0.737	0.003
CD16^hi^CD11b^lo^CD62L^br^ neutrophils						
Triglycerides	0.438	0.191	146	72.5	221	<0.0001
CD16^hi^CD11b^br^CD62L^lo^CXCR4^hi^ neutrophils						
Triglycerides	0.369	0.136	124	38.5	209	0.005

## Data Availability

The data used to support the findings of this study are available from the corresponding author upon request.
